# Methanol Production by “*Methylacidiphilum fumariolicum*” SolV under Different Growth Conditions

**DOI:** 10.1128/AEM.01188-20

**Published:** 2020-09-01

**Authors:** Carmen Hogendoorn, Arjan Pol, Guylaine H. L. Nuijten, Huub J. M. Op den Camp

**Affiliations:** a Department of Microbiology, Institute for Water and Wetland Research, Radboud University, Nijmegen, Netherlands; Kyoto University

**Keywords:** *Methylacidiphilum*, hydrogen, methane, methanol production, methanotroph

## Abstract

Industrial methanol production converts methane from natural gas into methanol through a multistep chemical process. Biological methane-to-methanol conversion under moderate conditions and using biogas would be more environmentally friendly. Methanotrophs, bacteria that use methane as an energy source, convert methane into methanol in a single step catalyzed by the enzyme methane monooxygenase, but inhibition of methanol dehydrogenase, which catalyzes the subsequent conversion of methanol into formaldehyde, is a major challenge. In this study, we used the thermoacidophilic methanotroph “*Methylacidiphilum fumariolicum*” SolV for biological methanol production. This bacterium possesses a XoxF-type methanol dehydrogenase that is dependent on rare earth elements for activity. By using a cultivation medium nearly devoid of lanthanides, we reduced methanol dehydrogenase activity and obtained a continuous methanol-producing microbial culture. The methanol production rate and conversion efficiency were growth-rate dependent. A maximal conversion efficiency of 63% mol methanol produced per mol methane consumed was obtained at a relatively high growth rate, with a methanol production rate of 0.88 mmol/g (dry weight)/h. This study demonstrates that methanotrophs can be used for continuous methanol production. Full-scale application will require additional increases in the titer, production rate, and efficiency, which can be achieved by further decreasing the lanthanide concentration through the use of increased biomass concentrations and novel reactor designs to supply sufficient gases, including methane, oxygen, and hydrogen.

**IMPORTANCE** The production of methanol, an important chemical, is completely dependent on natural gas. The current multistep chemical process uses high temperature and pressure to convert methane in natural gas to methanol. In this study, we used the methanotroph “*Methylacidiphilum fumariolicum*” SolV to achieve continuous methanol production from methane as the substrate. The production rate was highly dependent on the growth rate of this microorganism, and high conversion efficiencies were obtained. Using microorganisms for the production of methanol might enable the use of more sustainable sources of methane, such as biogas, rather than natural gas.

## Abstract (precis)

The production of methanol, an important chemical, is completely dependent on natural gas. The current multistep chemical process uses high temperature and pressure to convert methane in natural gas to methanol. In this study, we used the methanotroph “*Methylacidiphilum fumariolicum*” SolV to achieve continuous methanol production from methane as the substrate. The production rate was highly dependent on the growth rate of this microorganism, and high conversion efficiencies were obtained. Using microorganisms for the production of methanol might enable the use of more sustainable sources of methane, such as biogas, rather than natural gas.

## INTRODUCTION

Methane is an important energy source and chemical feedstock (1). It is the major component of both natural gas and biogas, the product of the anaerobic digestion of organic matter. The use of methane as an energy source or precursor in the chemical industry faces several challenges, including the transport of this gaseous compound. To create a more energy-dense and easy-to-transport chemical, methane can be converted into a liquid fuel such as methanol. The current chemical process for conversion of methane to methanol uses natural gas as the input; methane is first converted into syngas (CO + H_2_), which is subsequently converted into methanol. This catalytic process requires high temperatures (200 to 900°C) and pressure (5 to 20 MPa) (1). Compared with natural gas, biogas contains more impurities, such as CO_2_, NH_3_, and H_2_S, and thus is not directly suitable for the chemical methanol production process. Removing these contaminants is an energy-intensive and costly process (2).

Aerobic methanotrophs are microorganisms that grow on methane and conserve energy by oxidizing methane to CO_2_ using oxygen as a terminal electron acceptor (3, 4). The first step in the methane oxidation pathway is the conversion of methane into methanol catalyzed by the enzyme methane monooxygenase (pMMO or sMMO) (5). Under normal growth conditions, methanotrophs convert methanol into formaldehyde via the enzyme methanol dehydrogenase (MDH). Formaldehyde is then converted via formate into CO_2_, the final product of methane oxidation.

Aerobic methanotrophs belong taxonomically to *Alpha*- and *Gammaproteobacteria* and *Verrucomicrobia* (3, 6). Verrucomicrobial methanotrophs are extremophiles isolated from geothermal areas; they have a low optimal pH, and some isolates grow at high temperatures (7–10). These methanotrophs contain only the XoxF-type MDHs, which require a lanthanide as a cofactor, in contrast to the calcium-dependent MxaFI-type MDH (11, 12). Furthermore, verrucomicrobial methanotrophs use the Calvin-Benson-Bassham cycle for carbon fixation (13), and several species encode hydrogenases and can grow as Knallgas bacteria (14–16).

To date, biological methane-to-methanol conversion has only been studied in methanotrophs belonging to the *Alpha*- and *Gammaproteobacteria* that contain the MxaFI-type MDH (17–21). In order to obtain a methanol-producing microbial culture, the MDH activity is reduced by different MDH inhibitors, such as MgCl_2_ and EDTA (17). However, inhibition of MDH decreases ATP and reducing equivalent production. To compensate for this, formate can be added to serve as an extra electron donor (20, 22), but continuous methanol production has not been achieved.

In this research, biological methane-to-methanol conversion was investigated using “*Methylacidiphilum fumariolicum*” SolV, a species belonging to the phylum *Verrucomicrobia* (7). First, methanol production by *M. fumariolicum* SolV in cell suspensions and batch cultivation was investigated. The MDH activity was reduced by supplying the cells with medium depleted of lanthanides. Additionally, the effect of the addition of MDH inhibitors and electron donors, such as formate or hydrogen gas, on methanol production was investigated. Then, the effect of growth rate on methanol production was investigated in a phosphate-limited chemostat culture operated at different dilution rates, followed by an examination of the effect of ammonium or oxygen limitation on methanol production. Finally, the influence of lanthanide concentration on methanol production was determined in an oxygen-limited continuous culture.

## RESULTS

### Methanol accumulation using MDH inhibitors or hydrogen gas.

Methane-to-methanol conversion was first studied in batch incubations of cell suspensions of “*Methylacidiphilum fumariolicum*” SolV. Cells for these experiments were obtained from a phosphate-limited chemostat operated at a dilution rate of 0.025 h^−1^ and a stable low oxygen concentration (1% air saturation = 1.6 μM) and supplied with both methane and hydrogen gas. The medium contained only 20 nM cerium, and the residual cerium concentration in the bioreactor supernatant was below the limit of detection (<0.7 nM). The biomass was harvested by centrifugation and resuspended in 100 mM phosphate buffer, pH 3, followed by incubation of the suspension with methane at 55°C. Methanol production was not observed during these batch incubations. To test if increased MDH inhibition would stimulate methanol production, the cell suspensions were incubated with the presumed MDH inhibitors EDTA (1 mM) or MgCl_2_ (10 mM). However, these incubations did not result in methanol accumulation. In all batch incubations, methanol levels remained below the limit of detection (<0.1 mM) (Table S1 in the supplemental material).

This lack of methanol production might be caused by either insufficient MDH inhibition or a lack of ATP or reducing equivalents. Methane-to-methanol conversion requires the input of two electrons, and the required reducing equivalents are generated during the oxidation of methanol into CO_2_. The addition of extra electron donors other than methane might provide the required energy. Since *M. fumariolicum* SolV can oxidize hydrogen (14), the ability of hydrogen gas addition to support methanol production was tested. However, the addition of hydrogen gas, both with and without 1 mM EDTA, to the cell suspension did not result in methanol production (Table S1).

### Effect of formate and EDTA on methanol accumulation.

In a follow-up experiment, we tested if formate, an intermediate in the methane oxidation pathway, could provide the required reducing equivalents for methane-to-methanol conversion. Since formate has a pKa of 3.75, below this low pH formic acid is formed, which is highly toxic to *M. fumariolicum* SolV (7). Therefore, cells were grown in batch cultures in medium with a pH value of 5.5 and a nonlimiting cerium concentration (1 μM). The biomass from these cultures was harvested in the exponential phase by centrifugation and resuspended in buffer to an optical density at 600 nm (OD_600_) between 0.3 and 0.7.

**FIG 1:**
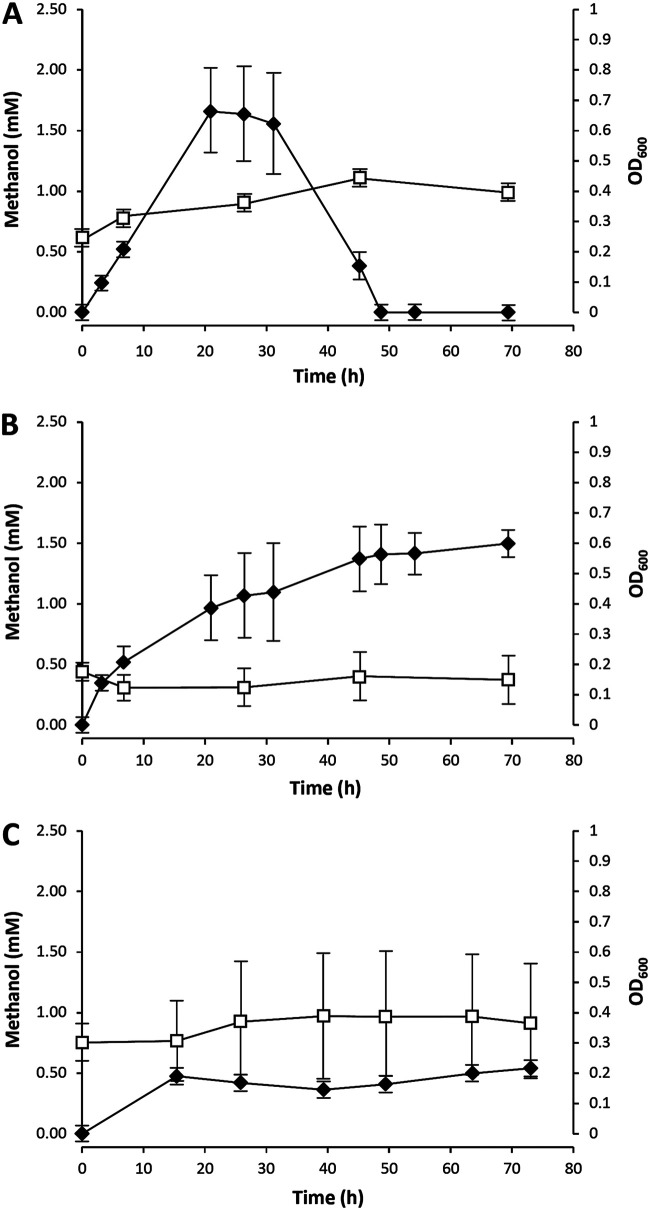
Methanol concentration (filled diamonds) and optical density at 600 nm (OD_600_) (open squares) during incubations in 100 mM phosphate (A), 100 mM phosphate and 20 mM formate (B), or 100 mM phosphate, 20 mM formate, and 1 mM EDTA (C). The values are the average of two experiments with the range of the independent values indicated.

During the incubations of these cell suspensions without formate, methanol was initially produced but subsequently fully consumed (Fig. 1A). The addition of 20 mM formate to the cell suspensions resulted in a stable final methanol concentration of 1.5 ± 0.1 mM (Fig. 1B), but the rate of methanol production was lower than that in the incubations without formate. Incubation of the cells with 20 mM formate and 1 mM EDTA resulted in a final methanol concentration of only 0.5 ± 0.1 mM (Fig. 1C), despite a lower biomass concentration compared with the other two incubation conditions. These results indicate that formate and/or EDTA might inhibit methanol production by *M. fumariolicum* SolV.

For all conditions, the highest increases in methanol production rates were obtained in the beginning of the incubation of the cell suspensions (Fig. 1). Interestingly, the optical density increased somewhat during these incubations, indicating biomass growth. This observation led to the hypothesis that growing cells may be required for methanol production. To test this hypothesis, additional batch cultivation experiments were performed.

### Methanol accumulation in batch cultivation.

In batch tests under normal growth conditions on methane, in which *M. fumariolicum* SolV was supplied with a cultivation medium containing 1 μM cerium, no measurable amount of methanol was produced, indicating that full oxidation of methane to carbon dioxide is not limited by MDH activity (Table 1). To reduce MDH activity, cerium was omitted from the cultivation medium in the next batch cultivation experiments. These batch experiments resulted in final methanol concentrations of 3.1 ± 0.7 and 2.0 ± 1.1 mM for cultivation at pH 3.0 and pH 5.5, respectively. These values were not significantly different. Interestingly, growth was not completely inhibited when cerium was omitted from the medium (Fig. S1), but an exponential increase in OD_600_ was not observed. Furthermore, the final OD of the suspension was lower (0.64 ± 0.13 and 0.33 ± 0.22 for pH 3.0 and pH 5.5, respectively) compared with batch cultivation in the presence of 1 μM cerium (0.93 ± 0.19 and 0.68 ± 0.02) (Table 1). These results suggested that trace amounts of lanthanides resulted in some MDH activity. To reduce the MDH activity even further, the cells were also incubated in the presence of 1 mM EDTA, but neither growth nor methanol production was observed (Table 1).

**TABLE 1:** Final OD_600_ and the final methanol concentration under different growth conditions^*a*^

Medium	pH	Gas composition	Final OD	Final methanol concn (mM)
1 μM cerium	3.0	10 v/v% CH_4_ + 5 v/v% CO_2_	0.93 ± 0.19	<0.05
No lanthanides	3.0	10 v/v% CH_4_ + 5 v/v% CO_2_	0.64 ± 0.13	3.1 ± 0.7
No lanthanides + 1 mM EDTA	3.0	10 v/v% CH_4_ + 5 v/v% CO_2_	0.04 ± 0.01	<0.05
No lanthanides	3.0	10 v/v% CH_4_ + 5 v/v% H_2_ + 5 v/v% CO_2_	0.20 ± 0.07	1.4 ± 0.7
1 μM cerium	5.5	10 v/v% CH_4_ + 5 v/v% CO_2_	0.68 ± 0.02	<0.05
No lanthanides	5.5	10 v/v% CH_4_ + 5 v/v% CO_2_	0.33 ± 0.22	2.0 ± 1.1
1 μM cerium + 20 mM formate	5.5	10 v/v% CH_4_ + 5 v/v% CO_2_	0.13 ± 0.00	<0.05
No lanthanides + 20 mM formate	5.5	10 v/v% CH_4_ + 5 v/v% CO_2_	0.24 ± 0.06	2.9 ± 0.4
20 mM formate	5.5	Air + 5 v/v% CO_2_	0.14 ± 0.02	<0.05

a Batch cultivation was performed for 90 h. The starting OD was 0.02 ± 0.01. The values are the average of three independent experiments ± standard deviation. The batch incubations that show methanol production did not differ significantly from each other. v/v%, percent by volume.

Next, the effect of the addition of hydrogen or formate as extra electron donors on methanol production was examined. Addition of hydrogen or formate resulted in 1.4 ± 0.7 mM or 2.9 ± 0.4 mM methanol, respectively, with no significant increase or decrease in the final methanol concentration compared to batches without addition (Table 1). To test whether *M. fumariolicum* SolV can oxidize formate, a batch incubation with 20 mM formate but without methane was performed. During this cultivation, the biomass concentration increased, indicating that *M. fumariolicum* SolV could oxidize and grow on formate, but the generated reducing equivalents were apparently not used for increased methanol production (Table 1).

These results indicated that methanol production is growth-rate dependent; however, the growth rate was challenging to control during these batch incubations. Lanthanide availability will influence the growth rate, final biomass concentration (11), and potentially the methanol production rate, but it was difficult to maintain a constant amount of lanthanides available for the biomass because the acidic medium could extract lanthanides from the glass bottles used for these incubations. The effect of growth rate on methanol production was therefore investigated in a steady-state chemostat culture operated at different fixed growth rates.

### Effect of growth rate on methanol production.

A phosphate-limited chemostat culture was established with methane and hydrogen as electron donors. In this system, biomass production was limited by available phosphate, and MDH activity was reduced by using only 20 nM cerium. Phosphate concentrations in the culture were around or below the detection limit (0.8 ± 0.3 μM). To test the effect of growth rate on methanol production, *M. fumariolicum* SolV was grown at a dilution rate of 0.0058 h^−1^, 0.014 h^−1^, 0.025 h^−1^, and 0.033 h^−1^. The dissolved oxygen concentration was maintained at a maximum air saturation of 1% (1.6 μM) to ensure that hydrogen oxidation was not inhibited by high oxygen concentrations (14).

**FIG 2:**
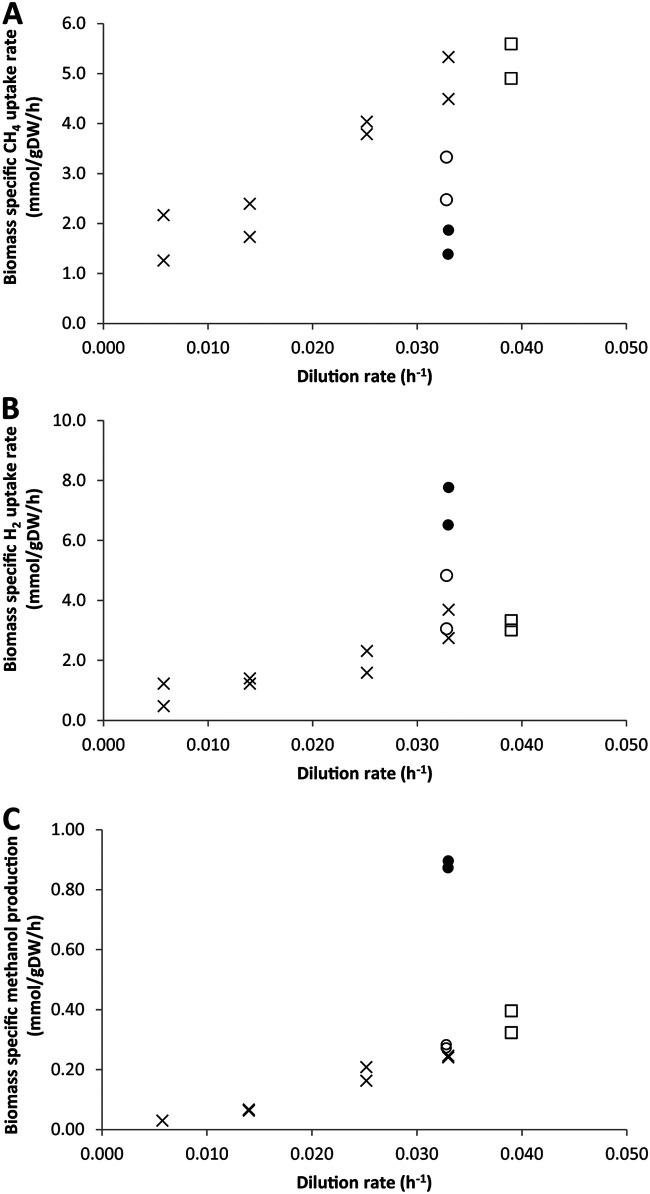
Biomass-specific methane uptake rate (A), biomass-specific hydrogen uptake rate (B), and biomass-specific methanol production rates (C) for chemostat cultures under different growth rates and substrate limitations. Shown are data for the PO_4_^3−^-limited chemostat fed with medium with 20 nM cerium (X symbol), NH_4_^+^-limited chemostat fed with medium with 20 nM cerium (open squares), O_2_-limited chemostat fed with medium with 20 nM cerium (open circles), and O_2_-limited chemostat without any cerium added to the medium (filled circles).

The highest methanol concentrations were achieved at the lowest growth rates. At the lowest growth rate of 0.0058 h^−1^, the methanol concentration reached 4.9 ± 0.4 mM, whereas at the high growth rate of 0.033 h^−1^ a methanol concentration of approximately 1.6 ± 0.0 mM was obtained (Table 2). Despite these lower concentrations, biomass-specific methanol production was highest at the highest growth rate, as there was the lowest biomass concentration. As shown in Fig. 2, a positive trend was observed between the growth rate and the biomass-specific methanol production rate. Thus, methanol production was growth-rate dependent.

**TABLE 2:** Biomass concentration, protein concentration, methanol concentration, and residual cerium concentrations under different growth rates and substrate limitations^*a*^

Growth rate (μ h^−1^)	*t*_d_ (h)	Limiting substrate	Biomass (g/liter)	Protein (mg/liter)	Methanol (mM)	Residual cerium (ppb)
0.0058	120	PO_4_^3−^	1.08 ± 0.03	366 ± 15	4.9 ± 0.4	<1
0.014	50	PO_4_^3−^	0.69 ± 0.07	231 ± 16	2.3 ± 0.1	<1
0.025	28	PO_4_^3−^	0.41 ± 0.07	160 ± 18	3.4 ± 0.3	<1
0.033	21	PO_4_^3−^	0.18 ± 0.03	104 ± 23	1.6 ± 0.0	<1
0.039	18	NH_4_^+^	0.22 ± 0.02	108 ± 5	2.8 ± 0.8	<1
0.033	21	O_2_	0.20 ± 0.03	82 ± 7	1.4 ± 0.3	<1
0.033	21	O_2_ without lanthanides^*b*^	0.13 ± 0.00	74 ± 3	4.1 ± 0.5	<1

a The values are the average of two experiments ± the range; *t*_d_, doubling time.

b Refers to the oxygen-limited chemostat cultures without any lanthanides added to the cultivation medium.

**FIG 3:**
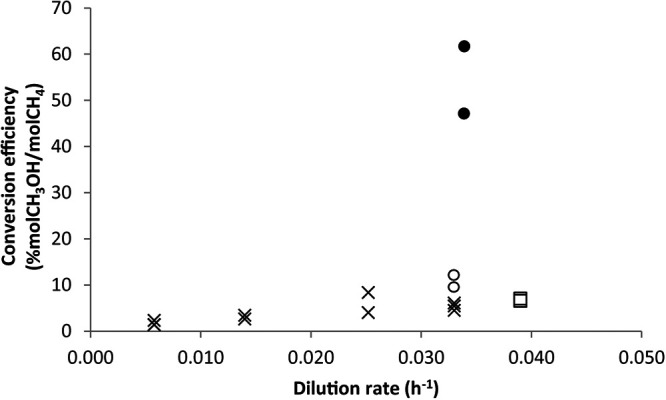
Methane-to-methanol conversion efficiency for chemostat cultures under different growth rates and substrate limitations. Shown are data for the PO_4_^3−^-limited chemostat fed with medium with 20 nM cerium (X symbols), NH_4_^+^-limited chemostat fed with medium with 20 nM cerium (open squares), O_2_-limited chemostat fed with medium with 20 nM cerium (open circles), and O_2_-limited chemostat without any cerium added to the medium (filled circles).

There was also a clear trend between the growth rate and conversion efficiency. The methanol yield on methane was highest at the highest growth rates, with conversion efficiencies of 6.2% ± 2.2% and 5.8% ± 0.3% at growth rates of 0.025 h^−1^ and 0.033 h^−1^, respectively (Fig. 3). At all growth rates, methane and hydrogen were consumed simultaneously. The biomass-specific uptake of both electron donors positively correlated with the growth rate (Fig. 2).

### PO_4_^3−^, NH_4_^+^, and O_2_ limitation.

The effects of different substrate limitations on methanol production were studied in a steady-state chemostat culture. The effects of ammonium and oxygen limitation were investigated separately in the reactor operating at a dilution rate of approximately 0.033 h^−1^ and supplied with medium containing only 20 nM cerium. In both cases, ammonium and oxygen concentrations were below the detection limits of 10 μM and 0.2 μM, respectively. Under these conditions, methanol was always produced, but the methanol concentration varied between 1.4 ± 0.3 and 2.8 ± 0.8 mM (Table 2). The phosphate-limited and ammonium-limited chemostat cultures had similar methane uptake rates, hydrogen uptake rates, methanol production rates, and conversion efficiencies. Under oxygen-limited growth conditions, hydrogen uptake increased, but the methane uptake rate decreased (Fig. 2). Interestingly, the biomass-specific methanol production rate remained similar, resulting in an increased yield of methanol on methane (Fig. 3). During oxygen-limited growth, approximately 9.8% of the consumed methane was excreted as methanol, indicating that >90% was still fully oxidized to CO_2_. To increase the conversion efficiency, MDH activity must be inhibited even further. Therefore, in the next set of experiments, an oxygen-limited culture was fed medium without added lanthanides.

### Cerium concentration.

During the oxygen-limited and lanthanide-depleted chemostat cultivation experiments, the biomass-specific methane uptake rate decreased while the biomass-specific hydrogen uptake rate increased compared with the ammonium- and phosphate-limited chemostat experiments fed 20 nM cerium (Fig. 3). Interestingly, the biomass-specific methanol production rate also increased under oxygen-limited and lanthanide-depleted growth conditions and reached 0.88 mmol/g (dry weight)/h. High conversion efficiencies of 48 and 63% (mol methanol/mol methane) were obtained at a methanol concentration of 4.1 ± 0.5 mM.

## DISCUSSION

### Methanol production using cell suspensions.

This study showed that methanol can be produced using growing cells of the verrucomicrobial methanotroph *Methylacidiphilum fumariolicum* SolV. Batch incubations of nongrowing cell suspensions at pH 3 did not produce methanol. Incubations at pH 5.5 resulted in methanol production, with the highest methanol production rates at the beginning of the incubation, during which a small increase in biomass was observed. Unless formate was added, the methanol was subsequently consumed by the suspension. This effect was also observed in *Methylocaldum* sp. (20), with oxidation of formate inhibiting the oxidation of methanol. No effect of presumed MDH inhibitors on methanol production was observed in cell suspensions of *M. fumariolicum* SolV. This is in contrast to studies using methanotrophs belonging to *Alphaproteobacteria* or *Gammaproteobacteria*. Previous studies using Methylosinus sporium, Methylosinus trichosporium, *Methylomonas* sp. DH-1, or *Methylocaldum* sp. reported methanol production using cell suspensions in phosphate buffer. Addition of EDTA, MgCl_2_, or formate resulted in higher methanol production rates (20), and final methanol concentrations of 4 to 30 mM methanol were obtained (19, 22, 23). Despite the fact that cell suspensions of *M. fumariolicum* SolV cannot be used for methanol production, we are convinced that the increased biomass-specific methanol production rate under oxygen-limited and lanthanide-depleted growth conditions in combination with the high conversion efficiencies (see below) supports the potential use of this methanotroph for methanol production.

### Methanol production in batch cultivation experiments.

During the incubations performed at pH 5.5, we observed a small increase in biomass concentration, leading us to hypothesize that growing cells are essential for methanol production. Batch cultivation in lanthanide-omitted medium resulted in a methanol-producing culture, but the increase in biomass suggested that MDH activity was not completely abolished. Most likely, lanthanides were transferred during inoculation or extracted by the acidic medium from the glass bottles used for these experiments, making it difficult to control lanthanide availability (11, 24). The concentration of lanthanides strongly influences the growth rate and therefore potentially the methanol production rate (11). This makes it challenging to study physiology and kinetics in these batch systems. To correlate the methanol production rate with the growth rate, we therefore used a chemostat cultivation approach.

### Methanol production is growth-rate dependent.

The effect of growth rate on methanol production was tested in a phosphate-limited chemostat culture supplemented with both methane and hydrogen as electron donors and lacking lanthanides. These experiments showed that the biomass-specific methanol production rate and conversion efficiency were positively correlated with the growth rate. The growth dependency of methanol production has not been systematically examined, but some studies have reported that methanol production rates are highest at the beginning of incubation (20, 22). Only a few studies have correlated the growth rate with the biomass-specific production rate, but many of these studies examined the formation of nonnative products by genetically engineered Saccharomyces cerevisiae, such as heterologous proteins or resveratrol (25, 26).

The methanol production rate was not affected by the different nutrient limitations, i.e., phosphate, ammonium, and oxygen. Different nutrient limitations might have different effects on intracellular metabolites, such as low levels of phosphorylated compounds, including ATP, under phosphate limitation, or reduced protein levels under nitrogen limitation (27). However, these different limitations and possible changes in intracellular metabolites did not greatly impact the biomass-specific methanol production rate in *M. fumariolicum* SolV. The nitrogen limitation was not alleviated despite the fact that *M. fumariolicum* SolV contains *nifDHK* genes and is capable of nitrogen fixation at low oxygen concentrations. In fact, the maximum growth rate under nitrogen-fixing conditions is 0.025 h^−1^, below the dilution rate set for the continuous cultures in the present study (28). It is not expected that increased methanol concentrations are caused by changes in expression, since *xoxF* gene expression thus far appeared to be constitutive and largely invariantly (Fig. S2).

The efficiency of methane-to-methanol conversion in *M. fumariolicum* SolV was dependent on the growth rate, applied nutrient limitation, and lanthanide concentration. During oxygen limitation, the methane uptake rate decreased, the hydrogen uptake rate increased, and methanol production was similar to that under phosphate and ammonium limitation. As a result, the conversion efficiency increased. Supplying the reactor with hydrogen is essential to ensure sufficient electron donors for growth and to minimize competition for reducing power between growth and product formation.

The highest obtained conversion efficiency was 63% mol_CH3OH_ · mol_CH4_^−1^. The rest of the methane was fully converted into CO_2_, since MDH activity was not completely inhibited. Most likely, the acidic medium still contained some lanthanides, resulting in residual MDH activity. Conversion efficiencies of 25% to 80% mol methanol/mol methane have been reported for cell suspensions of methanotrophic *Alphaproteobacteria*, *Gammaproteobacteria*, or consortia of these methanotrophs (17, 20, 29). During the cell suspension incubations in the present study, some MDH activity occurred, as a portion of the CH_4_ was fully oxidized to CO_2_. Whether MDH activity can be completely abolished remains unclear. The oxidation of methane into methanol requires two electrons, but the mechanism of electron transfer has not been resolved. There are three possible scenarios for electron transfer. First, NADH produced during formaldehyde or formate oxidation can be used as a reductant, while the electrons from methanol oxidation are used for ATP production. However, *M. fumariolicum* SolV does not encode a formaldehyde dehydrogenase, the enzyme that catalyzes the conversion of formaldehyde to formate, and this conversion route cannot provide electrons for methane-to-methanol conversion in this strain (7). The second scenario involves direct electron exchange between methanol oxidation and methane oxidation, whereby pMMO and MDH are coupled. However, if this were the case, methanol would not be excreted. The last possibility is that electrons from methanol oxidation are transferred through the ubiquinol pool by a reversibly operating ubiquinol-cytochrome-*c* reductase (30–32). In all of these possible electron transfer scenarios, part of the methane must be fully oxidized to CO_2_ in order to generate the electrons for methane-to-methanol conversion.

### Industrial application.

Methanol is an important chemical precursor and can be used as a chemical feedstock, a fuel, or in the denitrification process in wastewater treatment (33). Current chemical processes convert natural gas as input to methanol via a multistep process (1). Direct conversion of methane to methanol using methanotrophic bacteria is an interesting potential alternative that has low capital cost and can be performed at smaller scales compared to chemical methanol production processes (34). Methane is an inexpensive feedstock, which makes it attractive for microbial conversion into higher-value products (35). The most sustainable methane resource is biogas generated from organic waste. Biogas contains impurities, such as H_2_S, that could inhibit methanotrophs. To keep costs low, expensive gas cleaning procedures should be avoided, and thus methanotrophs that can tolerate relatively high H_2_S concentrations would be beneficial. *M. fumariolicum* SolV was enriched from a volcanic mudpot near Naples, Italy. These ecosystems emit harmful gases, including H_2_S (36), and it is likely that this microorganism can tolerate elevated concentrations of these gases in order to thrive in these geothermal areas. Initial experiments indicate active H_2_S oxidation (data not shown). Previously, “conventional” methanotrophs were shown to be inhibited by sulfide (37, 38).

Challenges in using aerobic methanotrophs for industrial processes include the gas-liquid transfer of CH_4_, O_2_, and potentially H_2_. These gases dissolve poorly in water, and intensive stirring requiring higher energy input would be needed to supply sufficient substrate, especially when high biomass concentrations are reached. Novel reactor designs with high gas-liquid transfer, such as U-loop fermenters designed for single-cell protein (SCP) production using the methanotroph Methylococcus capsulatus (39), could be an alternative to traditional stirred tanks. Suspended-growth membrane diffusion, pressurized bioreactors, and internal gas recirculation could also be used to increase the bio-availability of these poorly dissolvable gases (40).

There is increased interest in using extremophiles for the industrial production of bulk chemicals and biofuels (41). Methanotrophic *Verrucomicrobia* grow at low pH and moderate to high temperatures, characteristics that favor industrial applications (42). *M. fumariolicum* SolV grows at 55°C and has an optimal pH of approximately 3, which reduces the risk of contamination. Furthermore, the potential use of biogas as the substrate rather than natural gas makes this a sustainable process. Our research shows that the activity of the XoxF-type MDH can be reduced by removing lanthanides from the cultivation medium, thus generating a stable culture that converts methane to methanol with hydrogen as an additional electron donor. We achieved stable continuous production of 4.1 mM methanol with 0.13 g (dry weight) biomass/liter. To reach higher concentrations, the amount of biomass in the oxygen-limited chemostats could be easily increased by supplying more oxygen to the system.

In conclusion, this study used the verrucomicrobial methanotroph *Methylacidiphilum fumariolicum* SolV for the production of methanol. This methanotroph possesses an XoxF-type MDH that is dependent on rare earth elements for its activity. Supplying a cultivation medium without any lanthanides resulted in a high methanol production rate and efficiency. The methanol production was growth-rate dependent, and the highest methanol production rate and conversion efficiencies were achieved during oxygen-limited chemostat cultivation in which the biomass was supplied with both methane and hydrogen gas.

## MATERIALS AND METHODS

### Strains, media, and growth conditions of *M. fumariolicum* SolV.

*Methylacidiphilum fumariolicum* SolV was isolated from the Campi Flegrei volcanic region near Naples, Italy (7). Unless stated otherwise, the medium was composed of 0.2 mM MgCl_2_ · H_2_O, 0.2 mM CaCl_2_ · H_2_O, 1 mM Na_2_SO_4_, 2 mM K_2_SO_4_, 2 mM (NH_4_)_2_SO_2_, and 1 mM NaH_2_PO_4_ · H_2_O. The final trace element concentrations were 1 μM NiCl_2_ · 6H_2_O, CoCl_2_ · 6H_2_O, NaMoO_4_ · 2H_2_O, and ZnSO_4_ · 7H_2_O; 5 μM MnCl_2_ · 4H_2_O and FeSO_4_ · 7H_2_O; and 10 μM CuSO_4_ · 5H_2_O. In some experiments, CeCl_3_ · 6H_2_O was added to reach a final lanthanide concentration of either 20 nM or 1 μM. In this case, we added the needed amount of a stock solution of 100 mM CeCl_3_ · 7H_2_O to 20 liters of medium. The pH was adjusted to 3.0 or 5.5 by adding 1 M H_2_SO_4_ or 1 M NaOH.

### Batch cultivation.

To assess the effects of MDH inhibitors and the addition of an extra electron donor, 50 ml of culture from the chemostat operated at a dilution rate of 0.025 h^−1^ (see chemostat cultivation below) was harvested and centrifuged (5 min, 5,000 × *g*, 21°C). The pellet was resuspended in 50 ml of 100 mM phosphate buffer at either pH 3.0 or pH 5.5 and transferred into a 500-ml flask. To assess methanol production under growth conditions, 500-ml flasks containing 100 ml of medium were inoculated to an initial OD_600_ of 0.02. All flasks were sealed with red rubber stoppers. The headspace contained air, 10% CH_4_ (vol/vol), 5% CO_2_ (vol/vol), and optionally 5% H_2_ (vol/vol). The cultures were incubated at 55°C with shaking at 200 rpm.

### Chemostat cultivation.

For chemostat cultivation, the medium contained 20 nM cerium unless stated otherwise. For phosphate-limited chemostat cultivation, 50 μM NaH_2_PO_4_ · H_2_O was used. For ammonium limitation, the medium contained 1 mM (NH_4_)_2_SO_4_. Cultivation was performed in a 7-liter bioreactor controlled by in-Control (Applikon, the Netherlands) with a working volume of 5 liters. The temperature was 55°C and maintained using a heat blanket. The pH was measured by a pH electrode and controlled at 3.0 by addition of 1 M NaOH. The dissolved oxygen (DO) concentration was measured by a Clark-type oxygen electrode (Applikon, the Netherlands). The airflow was regulated to maintain a dissolved oxygen concentration of 1% air saturation unless stated otherwise. The reactor was stirred at 500 to 800 rpm using a stirrer with two Rushton impellers. The reactor was supplied with 70 ml/min CO_2_-argon (5%:95% [vol/vol]), 10 ml/min CH_4_-CO_2_ (95%:5% [vol/vol]), and 6 ml/min H_2_. For oxygen-limited chemostat cultivation, the airflow was set to 60 ml/min. The oxygen-limited chemostat cultivation without the lanthanide cerium was operated at an airflow rate of 40 ml/min.

### Optical density, dry weight, elemental analysis, and protein content.

The optical density was measured using a Cary 50 UV-VIS spectrophotometer (Agilent, Santa Clara, CA, USA). Dry weight (DW), carbon content, and nitrogen content were determined as described previously (14). Protein concentrations were measured using a Pierce bicinchoninic acid (BCA) protein assay kit (Thermo Fisher Scientific, Waltham, MA, USA).

### Gas composition.

Methane concentrations in the headspace of the bottles and the in- and outflow of the chemostat cultures were analyzed using an HP 5890 gas chromatograph (Agilent, Santa Clara, CA, USA) equipped with a Porapak Q column (1.8 m, inner diameter [ID] 2 mm) and a flame ionization detector. Hydrogen and carbon dioxide concentrations were measured using an HP 5890 gas chromatograph (Agilent, USA) equipped with a Porapak Q column (1.8 m, ID 2 mm) and a thermal conductivity detector. For both analyses, 100 μl of gas sample was injected. To determine oxygen consumption, 25 μl of gas was injected into an Agilent series 6890 gas chromatograph-mass spectrometer (GC-MS) and analyzed as described previously (43).

### Methanol and formate quantification.

The methanol concentration was determined colorimetrically using the 2,2’-azino-bis-(3-ethylbenzothiazoline-6-sulfonic acid) (ABTS) assay as described by Mangos and Haas but modified by dissolving the ABTS in 20 mM phosphate buffer, pH 7 (44). The formate concentration was determined as described by Sleat and Mah (45).

### Inductively coupled plasma mass spectrometry.

To determine the cerium concentration, 10 ml of clear supernatant was collected, passed through a 0.2-μm filter, and acidified with 65% nitric acid to reach a final concentration of 1%. After sample preparation, metal analysis was performed using an inductively coupled plasma mass spectrometer (ICP-MS, X series; Thermo Fisher Scientific, Waltham, MA, USA).

## Supplementary Material

Supplemental file 1
