# Supplementary material for: Methanol Production by “Methylacidiphilum fumariolicum” SolV under Different Growth Conditions
Source: Appl Environ Microbiol. 2020 Sep 1;86(18):e01188-20. doi: 10.1128/AEM.01188-20 (PMC7480378; doi:10.1128/AEM.01188-20)
Supplement: Supplemental file 1 [file AEM.01188-20-s0001.pdf]

Methanol production by *Methylacidiphilum fumariolicum* SolV under different growth conditions

Carmen Hogendoorn, Arjan Pol, Guylaine H. L. Nuijten and Huub J. M. Op den Camp

Supplementary Material

Table S1: Methanol production in 100 mM phosphate buffer pH 3.0 experiments.

Medium	Gas composition	pH	Final methanol (mM)
100 mM phosphate	10 v/v% CH_4 + 5v/v% CO_2	3	0 ± 0
100 mM phosphate, 1 mM EDTA	10 v/v% CH_4 + 5v/v% CO_2	3	0 ± 0
100 mM phosphate, 10 mM $MgCl_2$	10 v/v% CH_4 + 5v/v% CO_2	3	0 ± 0
100 mM phosphate	10 v/v% CH_4, 5v/v% H_2 + 5v/v% CO_2	3	0 ± 0
100 mM phosphate, 1 mM EDTA	10 v/v% CH_4, 5v/v% H_2 + 5v/v% CO_2	3	0 ± 0

The biomass was harvested from a cerium-limited chemostat, washed and resuspended in 100 mM phosphate buffer. The values are the average of three independent experiments.

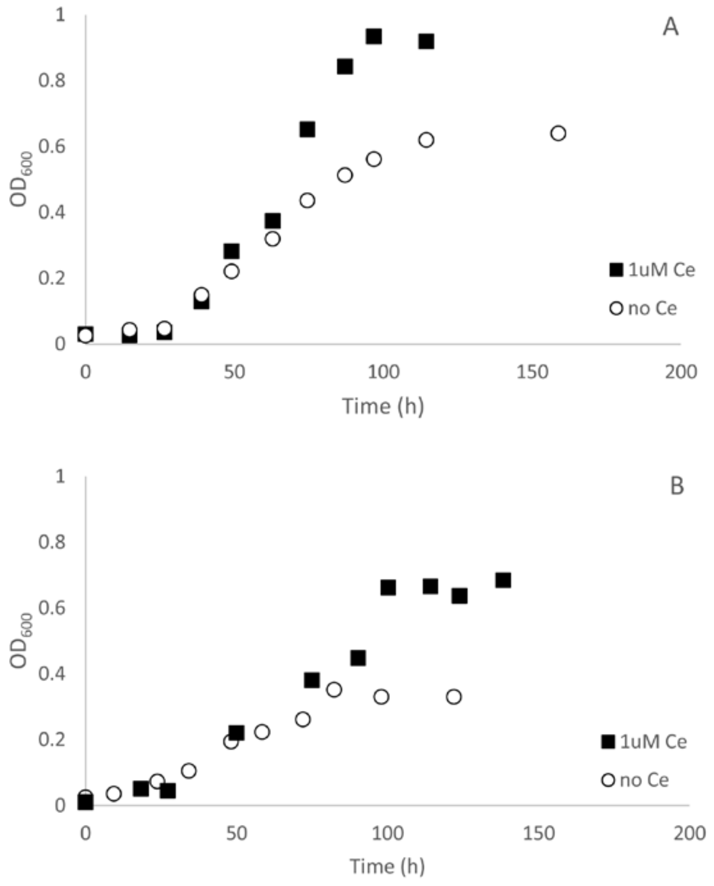

Figure S1: Biomass increase during batch experiments with cerium (1 µM) and without cerium performed at **A**. pH 3 or **B**. pH 5.5.

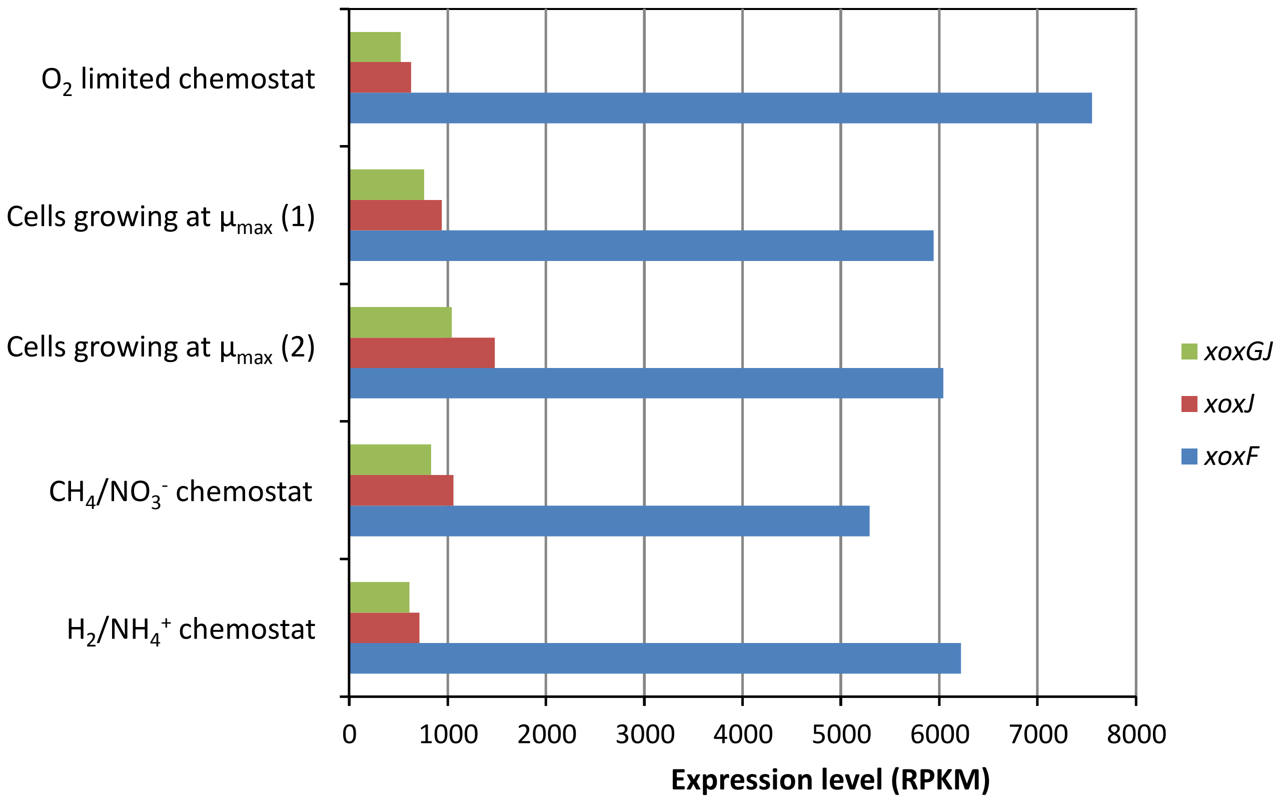

Figure S2: Expression levels (RPKM) of genes involved in methanol conversion in *M. fumariolicum* SolV. The *xoxF* gene encodes the methanol dehydrogenase (MDH); the *xoxJ* a MDH-associated periplasmic substrate binding protein and *xoxGJ* a fusion protein of the previous and the cytochrome c electron carrier. Data are from previous published work; Khadem et al. 2010 (1) and Mohammadi et al. 2016 (2).

References:

1. Khadem, AF, Pol A, Jetten MSM, Op den Camp HJM. 2010. Nitrogen fixation by the verrucomicrobial methanotroph Methylacidiphilum fumariolicum SolV. *Microbiology* **156**:1052-1059. doi:10.1099/mic.0.036061-0
2. Mohammadi SS, Pol A, van Alen TA, Jetten MSM, Op den Camp HJM. 2016. Methylacidiphilum fumariolicum SolV, a thermoacidophilic 'Knallgas' methanotroph with both an oxygen-sensitive and -insensitive hydrogenase. *ISME J* **11**:945-958. doi:10.1038/ismej.2016.171
